# Cre-LoxP and tamoxifen-induced deletion of ovarian quiescin sulfhydryl oxidase 2 showed disruption of ovulatory activity in mice

**DOI:** 10.1186/s13048-024-01388-2

**Published:** 2024-03-19

**Authors:** Shih-Yun Chen, Tse-En Wang, Wei-Yun Lee, Ya-Yi Yang, Hong-Chun Lai, Fuko Matsuda, Haruhiko Kosek, You-Tzung Chen, Sheng-Hsiang Li, Pei-Shiue Tsai

**Affiliations:** 1 Department of Veterinary Medicine, National Taiwan University, No. 1, Sec. 4, Roosevelt Rd., Taipei, 10617 Taiwan; 2 Graduate Institute of Veterinary Medicine, National Taiwan University, 10617 Taipei, Taiwan; 3 Research Center for Developmental Biology and Regenerative Medicine, National Taiwan University, 10617 Taipei, Taiwan; 4 Department of Veterinary Medical Sciences, Graduate School of Agricultural and Life Sciences, The University of Tokyo, Tokyo, 113-8657 Japan; 5 Center for Integrative Medical Sciences (IMS), RIEKN, Yokohama, Kanagawa 230-0045 Japan; 6 Department of Medical Research, MacKay Memorial Hospital, 25160 Tamsui, Taiwan; 7 Graduate Institute of Medical Genomics and Proteomics, College of Medicine, National Taiwan University, 10055 Taipei, Taiwan

**Keywords:** Oocyte development, QSOX2, Estrogen, Granulosa cells, Female reproduction

## Abstract

**Background:**

Quiescin sulfhydryl oxidase 2 (QSOX2) is a flavin adenine dinucleotide-dependent sulfhydryl oxidase that is known to be involved in protein folding, cell growth regulation, and redox state modification through oxidative activities. Earlier studies demonstrated the tissue and cellular localization of QSOX2 in the male reproductive tract, as well as the highly-regulated mechanism of QSOX2 protein synthesis and expression through the coordinated action of testosterone and epididymal-enriched amino acid, glutamate. However, the presence and the functions of QSOX2 in female reproduction are unknown. In this study, we applied the Cre-loxP gene manipulation system to generate the heterozygous and homozygous *Qsox2* knockout mice and examined its effects on ovarian function.

**Results:**

We demonstrated that QSOX2 was detected in the follicle-supporting cells (granulosa and cumulus cells) of ovarian follicles of all stages but was absent in the corpus luteum, suggesting its supportive role in folliculogenesis. In comparison with reproductive organogenesis in wild-type mice, there was no difference in testicular and epididymal structure in male *Qsox2* knockout; however, *Qsox2* knockout disrupted the regular ovulation process in female mice as a drastic decrease in the formation of the corpus luteum was detected, and no pregnancy was achieved when mating males with homozygous *Qsox2* knockout females. RNAseq analyses further revealed that *Qsox2* knockout altered critical signaling pathways and genes that are responsible for maintaining ovarian functions.

**Conclusion:**

Our data demonstrated for the first time that *Qsox2* is critical for ovarian function in mice.

**Supplementary Information:**

The online version contains supplementary material available at 10.1186/s13048-024-01388-2.

## Background

The mouse quiescin sulfhydryl oxidase 2 (QSOX2) is a 77 kDa enzyme from the quiescin Q6/FAD (flavin adenine dinucleotide)-dependent sulfhydryl oxidase (QSOX) family. Depending on the species of origin and its physiological functions, the QSOX2 protein exhibit various truncated forms. They are known to catalyze the formation of disulfide bonds in peptides and proteins through a catalytic activity: 2R-SH + O_2_ → R-S–S-R + H_2_O_2_ [1]. Due to their known chemical property, they are thought to be involved in various biological processes, such as protein folding, cell growth regulation, and redox state modification through oxidative activities [2], and dysregulation of this gene led to oxidative stress resulting in neurodegeneration [3]. Despite earlier identification of QSOX proteins in the reproductive tract by Chang et al*.* [4], their exact biological function in the reproduction field remained unclear until our previous studies showed specific epididymal distribution and sperm membrane association of QSOX2 [5, 6] and the specific testosterone-driven protein expression of QSOX2 in the mouse epididymis [7], suggested that QSOX2 is likely involved in supporting sperm development and maturation. Despite the fact that limited information regarding the roles of QSOX2 in the male reproductive tract has been shown, its expression or functional involvement in female reproduction is still unknown.

In the female reproductive system, the hypothalamic-pituitary–gonadal (HPG) axis acts as a master regulator, orchestrating and finely tuning folliculogenesis and oogenesis. The HPG axis consists of four major components, the hypothalamic gonadotropin-releasing hormone (GnRH), pituitary follicle-stimulating hormone (FSH), luteinizing hormone (LH), and the ovarian sex steroids. The pulsatile pattern of GnRH induces the pulsatile secretion of FSH and LH from the pituitary. In contrast, continuous release of GnRH will downregulate FSH and LH secretion due to prolonged exposure leading to desensitization [8, 9]. Ovarian steroidogenesis follows a cyclic pattern which includes a follicular phase, ovulation, and a luteal phase. During the follicular phase, dominant estradiol exerts negative feedback on FSH, and LH, suppressing GnRH through the kisspeptin neurons located [10, 11]. Furthermore, inhibin produced by granulosa cells (GCs) during the late follicular phase not only stimulates steroidogenesis but also exhibits a negative effect on FSH, but not LH [9]. By the time of estradiol surge, GnRH is released via the increase of kisspeptin, resulting in the LH surge leading to ovulation and luteinization [9]. Post-ovulation progesterone secretion by the corpus luteum (CL) becomes dominant in the luteal phase and negatively regulates estradiol effects on FSH, LH, and GnRH via increased GABA release [12].

The development of the oocyte is termed oogenesis. During the embryonic stage of the female, oogonia undergo mitosis to produce a population of primordial germ cells. These germ cells enter meiosis but are later arrested in the diplotene of prophase I [13]. When the primordial follicles enter initial recruitment, the oocytes grow in size. In addition, the zona pellucida (ZP) forms simultaneously around the oocyte, physically separating it from the adjacent GCs [14]. Trans-zonal cytoplasmic projections penetrating the ZP play a crucial role in the communication between the oocyte and cumulus cells, allowing the exchange of signal molecules and metabolites [15]. Once the follicle has reached the antral stage, the oocyte has grown in volume and has accumulated a significant amount of transcription factors, proteins, and organelles necessary for early embryo development [14]. Meiotic maturation occurs in response to the LH surge. The oocyte regains its meiotic ability by completing meiosis I before undergoing metaphase arrest of meiosis II.

As above-mentioned, we demonstrated earlier that QSOX2 is involved in supporting sperm maturation; therefore, in the current study, we aim to elucidate the role of QSOX2 in female reproduction, in particular, ovarian function.

## Results

### Cortical granule localization of ovarian QSOX2 exhibited specific cellular orientation at different follicular stages

**Fig. 1:**
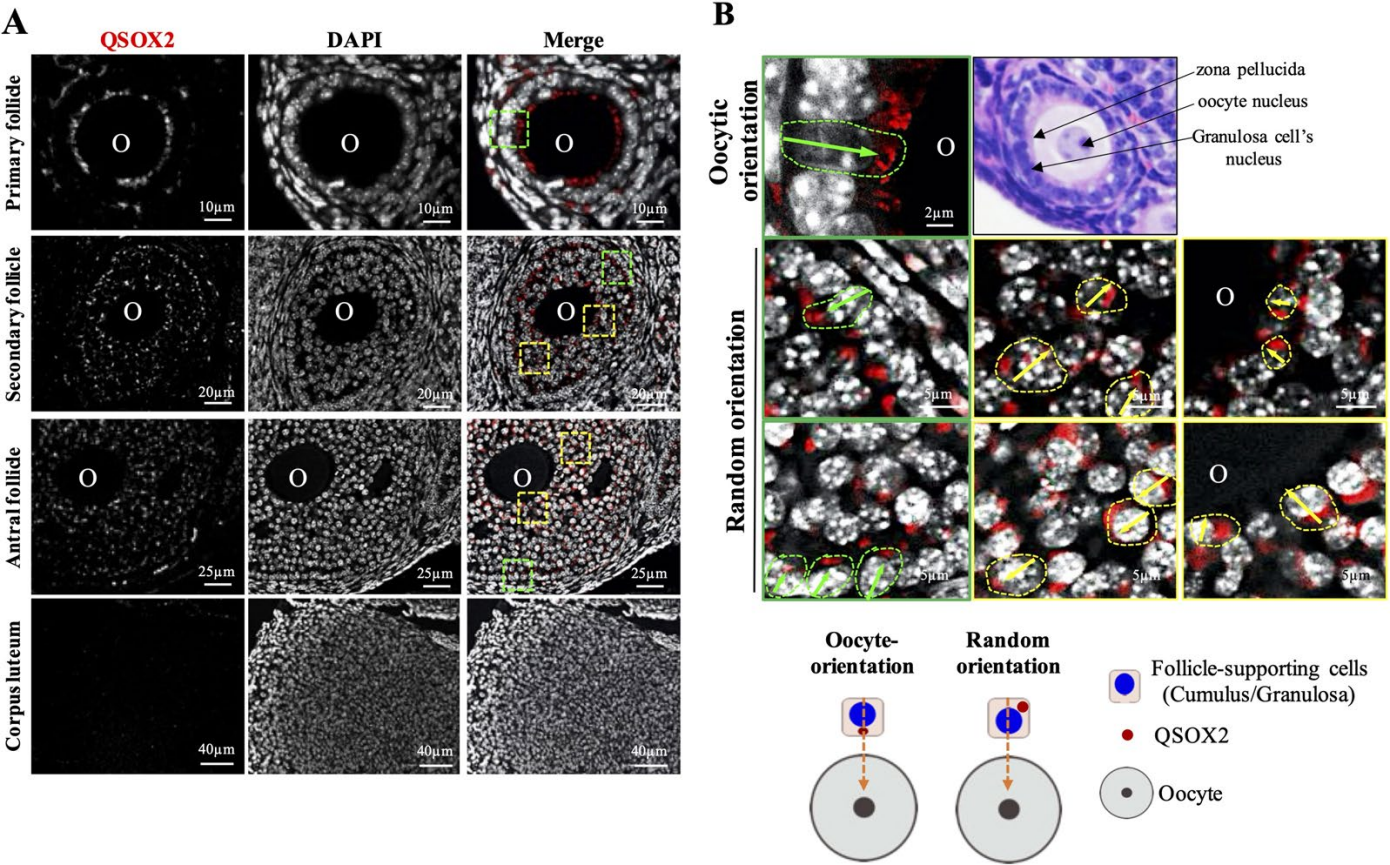
Cellular localization of QSOX2 protein in the mouse ovarian tissue. **A** Indirect immunofluorescent stating showed the presence of mQSOX2 (in red) in the granulosa cells of follicles of different stages, including primary, secondary, and antral follicles. However, no mQSOX2 was detected in the corpus luteum. **B** Magnified images showed two distinct mQSOX2 cellular localizations. Based on the hematoxylin–eosin stain, we showed that the mQSOX2 identified was located at the follicle-supporting cells rather than within the oocyte. The oocyte orientation, referred to as QSOX2, is located in a straight line between the oocyte and the granulosa cell. The random orientation, referred to as QSOX2 located randomly in the granulosa cells and is not in a straight line between the oocyte and the granulosa cell

IFA was utilized to determine the presence and the cellular localization of QSOX2 in the mouse ovary. We showed that QSOX2 was localized in the peri-nucleus region of GCs. As shown in Fig. 1A, QSOX2 signals were pronouncedly expressed at the peri-nuclear cellular localization of GCs of the ovarian follicles in the primary, secondary, and antral stages. Besides detecting QSOX2 signals in the follicles, we also detected weak signals in the interstitial regions of the above-mentioned follicular stages, indicating that QSOX2 is ubiquitously present in the ovary. In contrast to developing follicles, no specific signal can be detected in the corpus luteum (Fig. 1A). To distinguish whether the QSOX2 signal identified was located at the follicle-supporting cells (i.e. granulosa cells and cumulus cells), we examined follicles of all stages and showed that based on the localization of the zona pellucida (the outmost layer of the oocyte), the identified QSOX2 signals were observed in the follicle-supporting cells rather than within the oocyte (Fig. 1B). Apart from the presence of ovarian QSOX2; we also observed specific cellular orientation of GC-associated QSOX2. Two distinct QSOX2 orientations were defined: (1) oocyte-orientation: QSOX2 located in between the nucleus of GCs and the oocyte (green boxed); (2) random orientation: the presence of QSOX2 was not in a straight-line orientation between nuclei of GC and oocyte (yellow boxed) (Fig. 1B). As shown in Fig. 1B relatively dense QSOX2 signals were detected in an oocyte-oriented direction in the single-layer cuboidal GCs in the primary follicle, forming a ring-like structure around the oocyte (Fig. 1A, B, green boxed). In the secondary follicle, the outer-layered GCs featured strong QSOX2 oocyte-oriented signals forming a distinctive outer lining of the follicle (Fig. 1A, B, green boxed). In contrast to the unified oocyte orientation of QSOX2 at the outer layer of the GCs, QSOX2 in the mid-layers and inner layer of GCs surrounding the oocyte exhibited random orientation (Fig. 1A, B, yellow boxed). In the antral follicle, similar to primary and secondary follicles, QSOX2 signals from the outer-layered GCs showed oocyte orientation, and QSOX2 signals from the mid-layers and inner-layer GCs of the antral follicle showed random cellular distribution without specific orientation (Fig. 1A, B).

### Qsox2 gene deletion impaired the female fertilization capacity and ovarian functions in mice

**Fig. 2:**
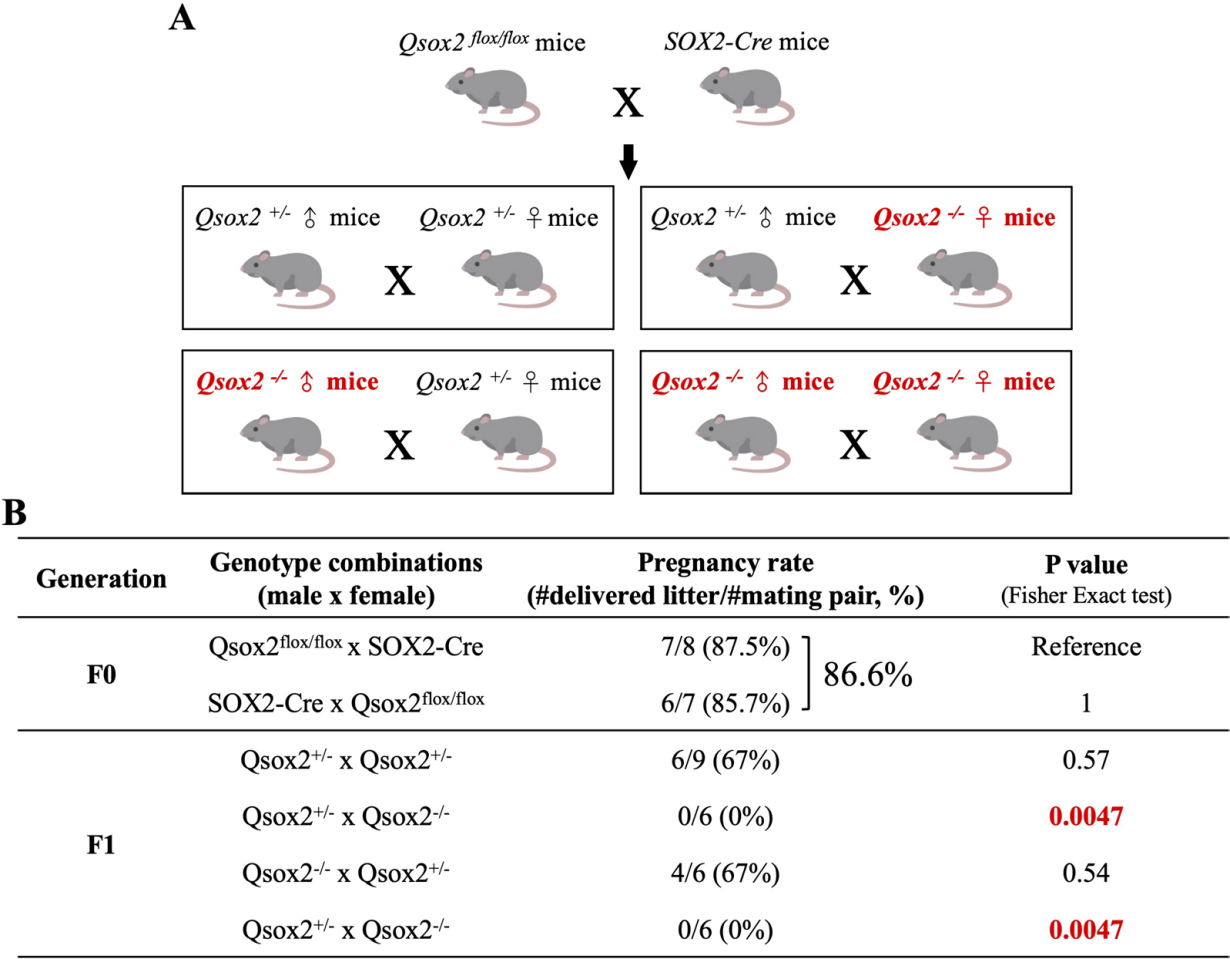
*Qsox2* deletion impaired breeding capacity in female mice. **A** To create *Qsox2* deletion, we bred *Qsox2* ^*flox/flox*^ mice with *SOX2*-Cre mice to generate conditional but universal *Qsox2* deletion. Later, four different genotype breeding pairs, including *Qsox2* ^±^ male x *Qsox2*^±^ *fe*male, *Qsox2* ^±^ male x *Qsox2* ^*−/−*^ *fe*male, *Qsox2* ^*−/−*^ male x *Qsox2* ^±^ *fe*male, *Qsox2* ^*−/−*^ male x *Qsox2* ^*−/−*^* fe*male were setup to examine the effects of *Qsox2* deletion on the breeding capacity **(B)** No apparent effects were detected in Qsox2^flox/flox^ bred with SOX2-Cre as 86.6% pregnancy rate can be achieved. A slight decrease in the pregnancy rate (67%) was detected when heterozygous females (*Qsox2* ^±^) were bred with either heterozygous male or homozygous *Qsox2* knockout males indicating that *Qsox2* knockout in males did not greatly affect the breeding capacity. In contrast, when homozygous *Qsox2* knockout females were involved, no pregnancy could be achieved. Fisher’s exact test indicated a significant decline (p value = 0.0047) in the pregnancy rate when compared with the F0 reference mating pair

To evaluate the breeding capacity of *Qsox2* gene deleted mice, we bred *Qsox2* ^*flox/flox*^ mice with *Sox2*-Cre mice to generate heterozygous *Qsox2* ^*flox/−*^ and further homozygous *Qsox2* ^*−/−*^ as described in the methods and material section (Fig. 2A). As shown in Fig. 2B, no apparent fertilization capacity disturbances were detected as 86.6% pregnancy rates were achieved when breeding of *Qsox2* ^*flox/flox*^ mice with *Sox2*-Cre mice. Interestingly, when breeding heterozygous *Qsox2* ^±^ male mice with heterozygous *Qsox2* ^±^ female mice or when breeding homozygous *Qsox2* ^*−/−*^ knockout male with heterozygous *Qsox2* ^±^ female mice, 67% pregnancy rates can still be achieved. However, when a homozygous knockout female mice were involved in breeding (either *Qsox2* ^±^ male mice bred with *Qsox2* ^*−/−*^ female mice or *Qsox2* ^*−/−*^ male mice bred with *Qsox2* ^*−/−*^ female mice), no successful pregnancy was recorded (Fig. 2B).

**Fig. 3:**
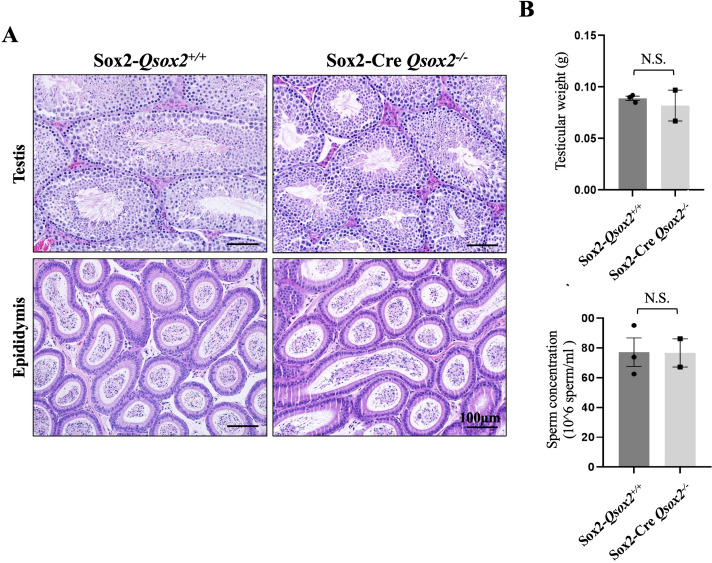
*Qsox2* deletion did not affect the testicular structure or sperm production in mice. **A** Histological analysis showed that compared with control mice (Sox-*Qsox2* ^+/+^), *Qsox2* knockout (Sox-Cre *Qsox2* ^−/−^) did not affect the testicular structure as the multi-layer spermatogenic structure was still visible in the testicular section, and the epididymal lumen (caput) was filled with sperm cells. **B** Testicular weight and sperm concentration did not differ between control and *Qsox2* knockout mice, supporting the notion that *Qsox2* knockout did not affect testis and sperm production in male mice. N.S.: non-significant

As shown in Fig. 3 that when compared with control male (Sox2-Cre *Qsox2* ^+/+^), *Qsox2* knockout male mice (Sox2-Cre *Qsox2* ^−/−^) showed normal testicular and epididymal structure with no significant differences in testicular weight, sperm production, and sperm concentration (Fig. 3). When sperm motility was evaluated, no differences between control and knockout mice were noted (data not shown).

**Fig. 4:**
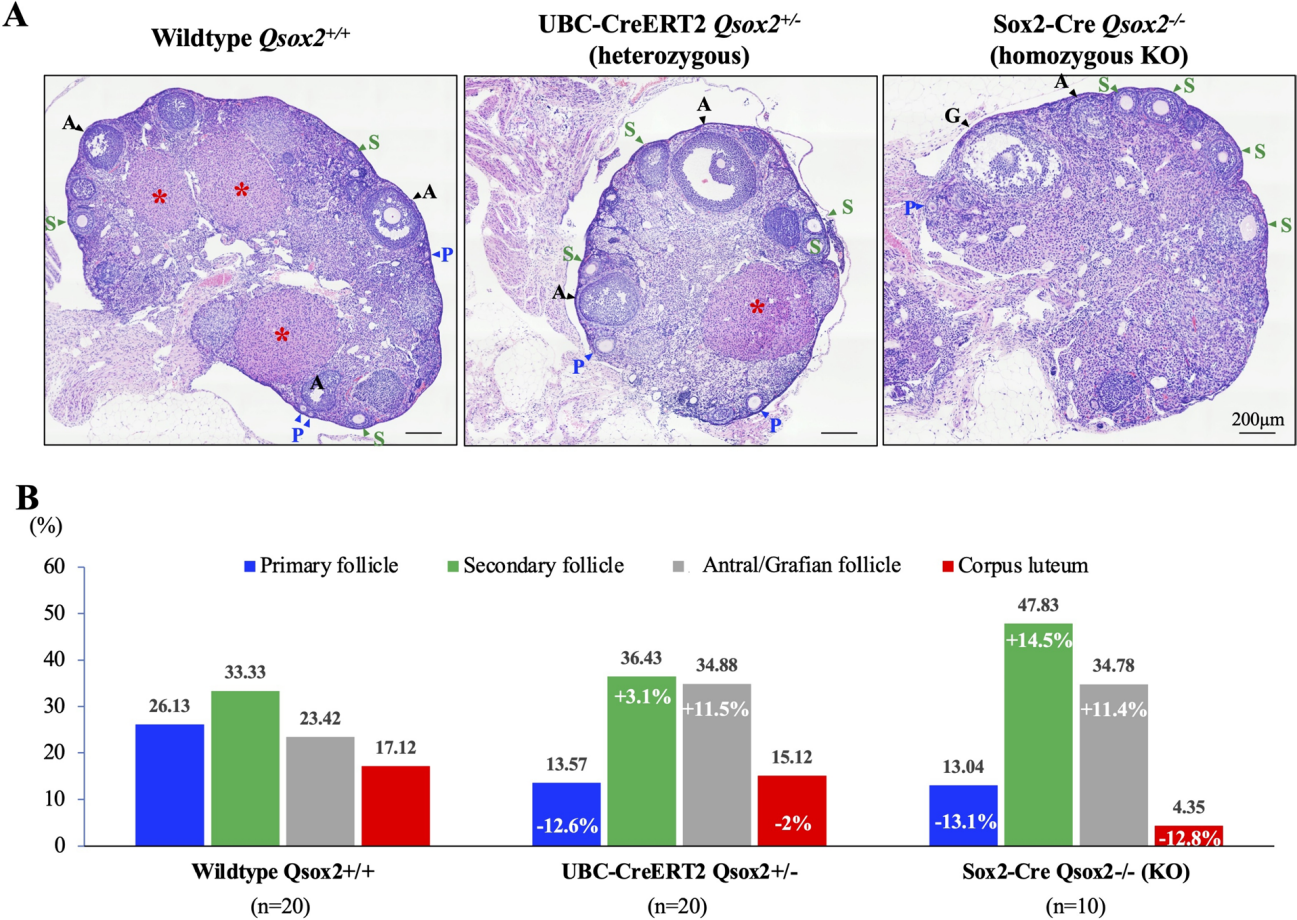
*Qsox2* deletion disrupted the homeostatic balance of the follicular cycle. **A** Hematoxylin and eosin stain of mouse ovaries showed the presence and a balanced ratio of follicles of different stages. In 20 control ovaries (from wild-type *Qsox2* ^+/+^) examined, the primary follicle (P), the secondary follicle (S), the antral follicle (A), and the corpus luteum (marked with asterisks) were all observed. In contrast, in both heterozygous (UBC-CreERT2 *Qsox2* ^±^) or homozygous knockout (Sox2-Cre *Qsox2* ^−/−^) ovaries, a sharp increase in the numbers of primary and secondary follicles with a drastic decrease in the number of corpus luteum were observed. **B** Quantification analysis showed a right-skewed distribution on the types of follicle in both heterozygous and homozygous knockout ovaries. In control ovaries, 26.1%, 33.3%, 23.4%, and 17.1% for the primary, secondary, antral follicle, and corpus luteum, respectively, were detected. However, when a single allele of *Qsox2* was deleted, an increased proportion of the secondary (40.9%, + 7.5% when compared with control ovary) and antral follicles (39.1%, + 15.7% when compared with control ovary) was observed; moreover, a sharply decreased percentage of corpus luteum (4.8%, -12.3% when compared with control ovary) was also detected. These changes in the balance of follicles of different stages were more apparent in homozygous *Qsox2* knockout ovaries

We, therefore, examined whether *Qsox2* knockout affects female fertility*.* When we examined the ovarian structure and the activity of mice with different genotypes, we observed that wild-type ovaries exhibited a relatively reasonable proportion of follicular and corpus luteum structure (26.13%, 33.33%, 23.42%, 17.12% for primary, secondary, antral/Graafian follicle, corpus luteum, respectively) indicating regular ovarian activity (Fig. 4). Surprisingly, when *Qsox2* gene was deleted, a skewed proportion of follicles was observed for both a single allele and total Qsox2 knockout. In those ovaries with *Qsox2* gene deletion, an apparent accumulation of secondary and antral/Graafian follicles was counted, and a minimal amount of corpus luteum was observed (Fig. 4B, 13.57%, 36.43%, 34.88%, 15.12% for primary, secondary, antral/Graafian follicle, and corpus luteum, respectively, in the heterozygous knockout ovary, and 13.04%, 47.83%, 34.78%, 4.35% for primary, secondary, antral/Graafian follicle, corpus luteum, respectively in the homozygous knockout ovary). Taken together, our data showed that *Qsox2* gene deletion impaired the mouse's fertilization capacity and ovarian functions.

### Qsox2 gene deletion altered overall ovarian genes expression and signaling pathways associated with ovarian functions

**Fig. 5:**
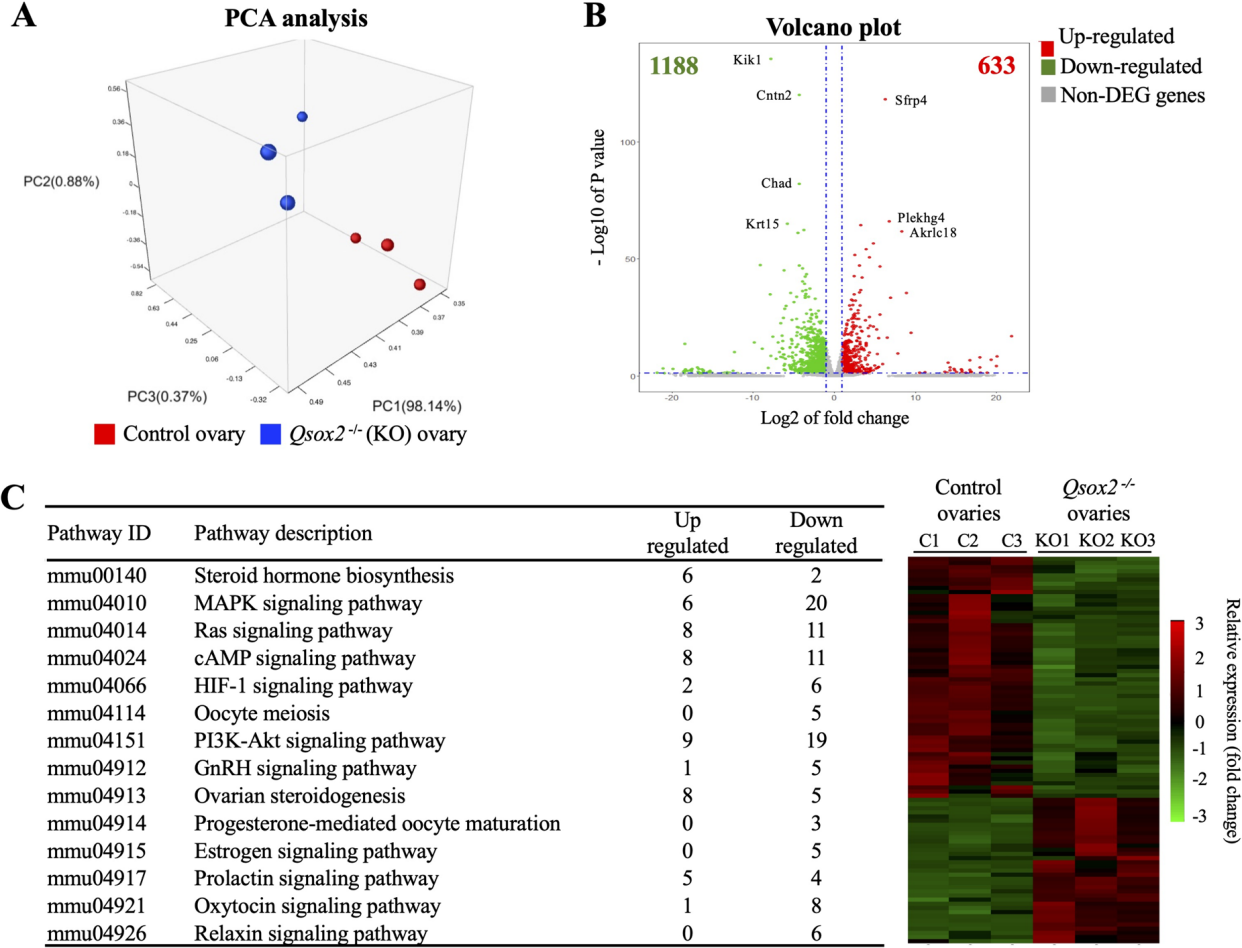
RNAseq analysis revealed ovarian genes and signaling pathways affected by Qsox2 deletion. **A** The principal component analysis (PCA) presents the two distinct group distributions of the control and KO ovaries with a stress value of 1.0*10^–5^. **B** Volcano plot of differentially expressed genes (DEGs) in the ovaries of *Qsox2* KO mice. A total of 1821 differentially expressed genes (DEGs). 633 up- and 1188 down-regulated genes are indicated as red and green dots, respectively (fold change ≥ 2 and *P*-value < 0.05). **C** Selected signaling pathways and its associated heatmap showed the relative expression patterns of the 164 DEGs (54 up- and 110 down-regulated genes) between control and *Qsox2* KO groups. The color scale of the heatmap ranges from green (down-regulation) to red (up-regulation)

The overall systemic gene expression changes were first analyzed using next-generation sequence by comparing wild-type with Sox2-Cre *Qsox2* ^−/−^ knockout mice (total knock out). As shown in Fig. 5A, the principal component analysis (PCA) showed a clustered overall gene profile with high similarity within ovarian samples from wild-type (red circles) and *Qsox2* ^−/−^ knockout mice (blue circles). Moreover, significant differences between wildtype *Qsox2* ^+*/*+^ and homozygous knockout *Qsox2* ^−/−^ ovaries were detected via nonmetric multidimensional scaling (NMDS) analysis with a stress value of 1.0*10^–6^. We identified 633 up-regulated genes and 1188 down-regulated genes that are differentially expressed genes after *Qsox2* deletion, indicating that the ovarian activity of *Qsox2* knockout mice was significantly altered in comparison to the control group (Fig. 5B).

Using a fold change cutoff of 2 and a *P*-value cutoff of 0.05, we further identified 14 signaling pathways, including 54 up-regulated genes and 110 down-regulated genes that were significantly affected by the deletion of the *Qsox2* and were related to ovarian activities (e.g., folliculogenesis, oogenesis, and hormone regulation) (Fig. 5C). As shown in Fig. 5C (right panel, heatmap), the DEG clusters that are up-or down-regulated in *Qsox2* knockout mice were significantly different from the wildtype group indicating that ovarian activity of *Qsox2* knockout mice was significantly affected.

### Qsox2 gene deletion altered genes expression related to functions required for regular ovarian activities

**Fig. 6:**
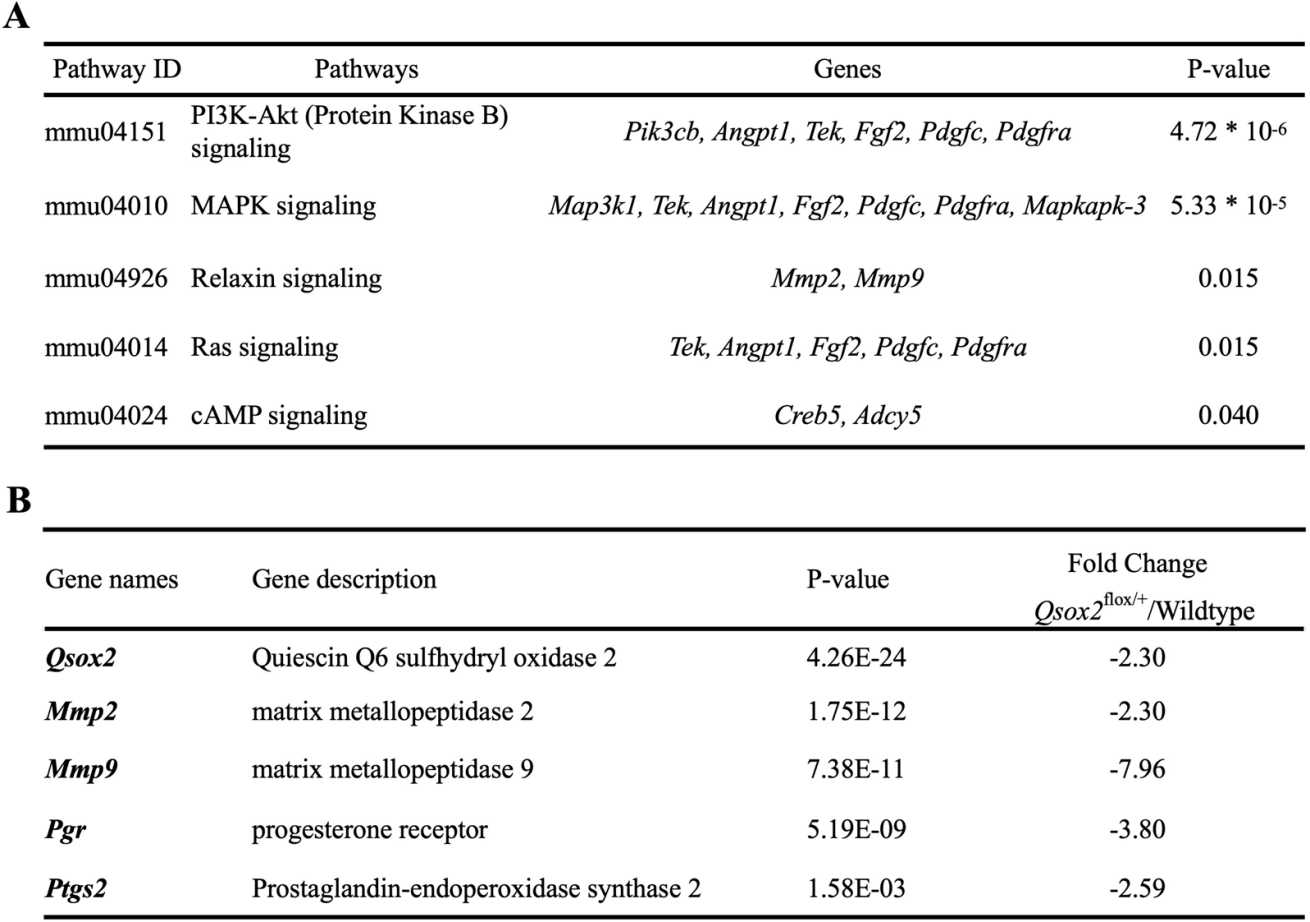
Relevant signaling pathways maintaining ovarian activities and ovulation process. **A** Five significantly affected pathways (*P* value < 0.05) in the *Qsox2* KO mouse model and their related genes of interest. **B** Critical genes that are essential for regulating the ovulatory process

In Fig. 6A, cyclic adenosine monophosphate (cAMP), hypoxia-inducible factor 1 (HIF-1), mitogen‑activated protein kinase (MAPK), phosphatidylinositol 3' -kinase (PI3K)-ATK, Ras, and Relaxin signaling pathways showed the highest significance after *Qsox2* gene deletion. Of particular interest, within these 6 signaling pathways, we identified 2 potential genes that can be regulated by estradiol (*Pik3cb, Map3k1*), 3 were known to regulate the production of cAMP and its pathway (*Creb5, Adcy5, Mapkapk-3*), 5 were related to angiogenesis (*Angpt1, Tek, Fgf2, Pdgfc, Pdgfra*). Moreover, including *Qsox2*, genes that were already known to be responsible for ovulation, such as matrix metallopeptidase 2 (*Mmp2*), matrix metallopeptidase 9 (*Mmp9*), progesterone receptor (*Pgr*), prostaglandin-endoperoxidase synthase 2 (Ptgs2) were significantly and highly down-regulated in the ovary of *Qsox2* knockout mice (Fig. 6B). All above-mentioned evidence supported the fact that *Qsox2* gene deletion altered genes expression related to functions required for regular ovarian activities and may partially explain the observed disruption of regular ovulation activity (Fig. 4).

## Discussion

Despite the fact that QSOX2 was identified in 2003, its function and the role of QSOX2 in reproduction are still largely unknown. Our results demonstrated for the first time that mouse ovarian QSOX2 was located in the follicle-supporting cells (i.e. GCs and cumulus cells), and *Qsox2* gene deletion compromised the ovulation and the subsequent CL formation. Moreover, various genes and essential signaling pathways that were known to be associated with ovarian activities were downregulated. These findings supported our hypothesis that QSOX2 might be involved in regulating ovarian activity.

GCs acquire rapid growth ability in response to FSH and estradiol exposure, resulting in the formation of large preovulatory follicles from the primary stage [16]. However, in the mature CL, these luteal cells have exited the cell cycle of proliferation [17]. This echoes the observation in our study that QSOX2 expression was detected in GCs but not in the CL, providing strong evidence that QSOX2 is also involved in regulating cell proliferation and growth. The oocyte orientation of the QSOX2 signal in the outer layer of GCs may suggest creating an encircled microenvironment ideal for folliculogenesis during the primary to the antral stage. The random orientation of QSOX2 expression in the other layers might indicate that QSOX2 responds to estradiol signaling in a non-specific direction. However, more experimental evidence will be needed to support this speculation.

Earlier studies showed that the PI3K-AKT pathway is the critical signaling pathway in promoting GCs proliferation, differentiation, controlling ovulation, and forming CL while inhibiting follicular atresia during folliculogenesis [18, 19]. The MAPK pathway is also known to stimulate GC proliferation and support the development and maturation of oocytes [20]. Both PI3K-AKT and MAPK pathways can be activated by estradiol through G-protein coupled estrogen receptors to promote cell proliferation and survival [21]. In our study, QSOX2 expression was positively correlated with estradiol fluctuation during the estrus cycle (data not shown). In *Qsox2* gene deleted mice, genes associated with estrogen signaling pathways were down-regulated, and both PI3K-AKT and MAPK pathways were significantly affected (39 genes were down-regulated), suggesting a close association between estradiol-QSOX2 interaction in the regulation of PI3K-ATK and MAPK pathways.

An earlier study showed that LH mediates the activation of adenylyl cyclase, thereby increasing cAMP concentration in GCs [22]. LH surge also stimulates the PI3K-AKT directly and through the activation of the Ras signaling cascade. In addition, the Ras pathway, promoted by cAMP, mediates the PI3K-AKT pathway and stimulates the MAPK pathway [23]. These signaling cascades are crucial for ovulation, luteinization, cumulus-oocyte complexes (COCs) expansion, and oocyte maturation under LH surge. Our study revealed a significant decrease in the numbers of CL formation in both single *Qsox2* allele deletion of total *Qsox2* KO mice; therefore, it is likely that *Qsox2* deletion down-regulated genes (in total 61 genes were down-regulated) and signaling pathways (PI3K-AKT, MAPK, Ras, cAMP) that are responsible for maintaining regular ovulation, and led to a significant reduction of CL in the mouse ovaries. Interestingly, deleting a *Qsox2* gene also resulted in the down-regulation of Creb5 and Adcy5, located in the cAMP signaling pathway. The Adcy5 gene encoding the protein adenylyl cyclase V is a critical factor for cAMP production. The cAMP-response element-binding (CREB) protein 5 encoded by the Creb5 gene is downstream of cAMP, mediating various gene transcriptions. In addition, a previous study has shown that the MAPK-activated protein kinase 3 (MAPKAPK3) downstream of the MAPK cascade also seems to maintain CREB phosphorylation during luteinization [24]. Therefore, the downregulated *Creb5* and *Mapkapk-3* genes in QSOX2 KO mice imply a negative effect on ovulation and CL formation due to defective cAMP production.

It is worth mentioning that angiogenesis plays a critical role in forming the CL. The GC layer in the follicles remains avascular throughout folliculogenesis. However, following the LH surge, the breakdown of the basement membrane allows endothelial cells and pericytes to invade and vascularize the luteinizing GCs. Up to 85% of the proliferating cells in the developing CL are of vascular origin in this rapid and intense process of angiogenesis [25]. Several genes, including *Angpt1, Tek, Fgf2, Pdgfc, and Pdgfra,* related to angiogenesis during luteinization, were also downregulated in our *Qsox2* KO mouse model. An increase of fibroblast growth factor 2 (FGF2) in GCs by LH surge stimulates the migration of endothelial cells and activates fibroblasts during early luteinization [26]. The platelet-derived growth factor (PDGF) also plays an essential role during the follicular–luteal transition in mice; moreover, the expressions of PDGFRA (PDGF receptor A) and its ligands, PDGFC, are both found in the GCs of preovulatory follicles and luteal parenchymal cells [27], suggesting their importance in the micro-vascularization in luteinizing GCs. Similarly, an early publication showed that before the LH surge, angiopoietin 1 (ANGPT1) and its receptor endothelial-specific tyrosine kinase (TEK) are essential for the vascular maturation of preovulatory follicles. After the LH surge, ANGPT1 can also promote new vessel formation in the CL, resulting in fully vascularized luteal cells [28]. However, in the ovary obtained from the *Qsox2* KO mouse, the above-mentioned genes essential for forming the CL were down-regulated. Taken together, these pieces of evidence suggest that the downregulation of *Angpt1, Fgf2, Pdgfc,* and *Pdgfra* genes in QSOX2 KO mice likely contribute to the impairment in angiogenesis that is required for CL formation.

Apart from CL vascularization, relaxin-mediated breakdown of the extracellular matrix in the granulosa and thecal cell compartment is equally critical to CL formation and ovulation [29]. The matrix metalloproteinases (MMPs), progesterone receptor (*Pgr*), and prostaglandin-endoperoxidase synthase 2 (*Ptgs2*) are known to involve in this process in response to LH surge. In particular, the mouse ovaries contain MMP2 (also known as type IV collagenase) and MMP9 (also known as type V collagenase) to break down the basement membrane collagen type IV and V, allowing rupture of the follicle and extrusion of the oocyte [30]. Our study observed the down-regulation of MMP2 and MMP9 in the relaxin pathway, suggesting that *Qsox2* deletion may inhibit ovulation due to defected follicle rupture extrusion and extrusion required upon normal ovulation process. Altogether, the *Qsox2* gene may directly or indirectly regulate ovulation, angiogenesis, and CL formation via the above-mentioned genes and signaling pathways. To our knowledge, this is the first study investigating the expression and potential biological function of QSOX2 in ovarian activity.

One interesting phenomenon worth mentioning is the changes in ovarian steroids in the QSOX2 KO female; even though a limited amount of serum was obtained, serum estrogen concentration showed no differences between wildtype (28.3 ± 6.4 pg/ml) and QSOX2 KO (30.7 ± 8.3 pg/ml) female. When we further reviewed genes that are associated with ovarian steroids, we saw no statistical differences between wildtype and QSOX2 KO females on gonadotropin-releasing hormone (GnRH, p = 0.87), follicular stimulating hormone (FSH, p = 0.80), luteinizing hormone (LH, p = 0.41), oxytocin (p = 0.98), these suggested that conditional, but systemic QSOX2 KO in the current study did not alter the function of ovarian steroids in the hypothalamus or anterior pituitary gland. Another interesting finding is that inhibin, a sex steroid hormone secreted by the matured follicle to negatively regulate the secretion of FSH, LH was upregulated. This suggested that the ovary and follicles from the QSOX2 KO female can respond normally to the FSH (evident from the observation of follicles of all stages), and can function normally (evident from the normal estrogen level and upregulation of inhibin).

## Conclusions

QSOX2 may act as a regulatory factor of GC proliferation under the influence of estradiol. In addition, QSOX2 may also promote GC luteinization by mediating ovulation and angiogenesis in response to LH surge, and QSOX2 knockout compromises the above-mentioned ovarian activities. Despite the fact that infertility observed in the current study may contribute, in part, by indirect effects from other tissues since the SOX2 gene is expressed in many tissues (e.g. brain and uterus) other than the ovary. However, we observed no apparent differences in the histology of the uterus between the wild-type and *Qsox2-/-* mice (supplementary Fig. 2), which may indicate the potential contribution of the uterus to infertility. Future studies with different tissue-specific Cre mice might be required to dissect the contribution of QSOX2 from different tissues to female fertility.

## Methods

### Chemicals, reagents, antibodies

All chemicals were obtained from Sigma-Aldrich (St. Louis, MO, USA). For primary antibodies, rabbit polyclonal anti-QSOX2 (#Ab121376) was purchased from Abcam (Cambridge, UK). Secondary antibodies conjugated with fluorophore were acquired from Jackson ImmunoResearch Laboratories Inc. (West Grove, PA, USA). Corn oil (#C8267) and tamoxifen (#T5648) were obtained from Sigma-Aldrich. Tamoxifen was dissolved in 100% corn oil at 56ºC overnight with a periodic vortex. After dissolution, the solution was stored at 4ºC for no more than a week before use.

### Animals

Animals were obtained from BioLASCO, Taiwan Co., Ltd. and were monitored daily by a certified veterinarian. For the experiment regarding the relation between QSOX2 protein expression and the estrus cycle, 10-week-old female C57BL/6 J mice were used. To evaluate the effects of *Qsox2* gene deletion on female ovarian activity, wildtype, SOX2-Cre, UBC-CreERT2, and *Qsox2* ^flox/+^ mice were used for total or conditional knock-out experiments. Production of genetically modified animals was followed as previously described. *Qsox2* ^flox/+^ (RBRC10218) mice were generated by RIKEN Institute (Wako, Saitama, Japan). SOX2-Cre and Tg (Ubc-CreERT2) [31] mice were kindly provided by Professor You-Tzung Chen and Shuei-Liong Lin, respectively, from National Taiwan University Hospital, Taiwan. All animals were housed in a temperature-controlled environment under a 12/12 h controlled light–dark cycle with ad libitum access to food and water throughout the study. Animal handling and experimental procedures were carried out under the regulation and permission of Institutional Animal Care and Use Committee (IACUC) protocols at the National Taiwan University (NTU-108-EL-00125, NTU-109-EL-00076, Taiwan).

### Estrus cycle determination

Estrus cyclicity was monitored daily over 16 consecutive days (covering 4 estrus cycles) by performing a vaginal smear and cytological examination between 12:00 PM and 2:00 PM. Vaginal lavage was performed to collect the vaginal epithelium for examination. In short, 100 μl of phosphate-buffered saline (PBS) was gently flushed into the vaginal canal, the lavage was collected and smeared onto glass slides, and collected cells were stained with Liu’s stain (#03R011, ASK, Taiwan). Based on general cytology categorization, the proestrus stage was defined by predominant nucleated epithelial cells, the estrus stage was determined by the presence of a majority of cornified squamous epithelial cells, the metestrus stage is characterized by the presence of both cornified squamous epithelial cells and leukocytes, and mostly leukocytes were observed at diestrus stage. Once the estrus cycle of the animal was determined, three animals in each estrus stage were sacrificed, and ovaries were collected for either indirect immunofluorescent staining or were flash-frozen with liquid nitrogen and stored at -80 °C for later use.

### Induction and genotype validations of QSOX2 knockout animals

*Qsox2* flox animals (single allele) were generated by Dr. Kosek’s lab at RIKEN Institute (Japan) and were kindly given as research collaboration materials. Tamoxifen inducible QSOX2 transgenic mice were obtained from inbred crosses of *Qsox2* ^flox/flox^ mouse with Tg (Ubc-CreERT2) mouse. Transgenic mice with Ubc-CreERT2 genotype were randomly allocated into vehicle control (n = 5) and tamoxifen-induction group (n = 8). Mice from the latter group were given 0.05 mg/g body weight tamoxifen daily via intraperitoneal (IP) injection for consecutive 5 days for two rounds once they reached 5 weeks old. To determine the efficacy of tamoxifen-induced gene deletion, the genotype of UBC-CreERT2 *Qsox2* ^flox/+^ mice was assayed by poly chain reaction (PCR) before and after tamoxifen injections. The toes of the mice were cut and placed into 300 μL NaOH at 100ºC for 1.5 h for genomic DNA extraction. Twenty-five μL TRIS was added to each sample and all the samples were centrifuged at 14,000 G at 4ºC for 10 min. PCR mixture was prepared using 1 μL of the supernatant DNA extract and 0.4 μL 10 μM primer pair 1 (5’ to 3’, forward: CAGCAGGGTCTCAGGTAGTTC; reverse: AGAACCAGGATGTGTGCCG). The PCR program (95ºC, 3 min., 95ºC, 30 s., 60ºC, 30 s., 72ºC, 1 min., 72ºC, 5 min, 35 cycles) was used for all of the samples, and agarose gel electrophoresis was utilized for DNA separation and the later result visualization (supplementary Fig. 1A). Once the genotype of the mice was determined, female C57BL/6 J mice (genotype: UBC-CreERT2 *Qsox2* ^flox/+^) and control wildtype *Qsox2* ^+/+^ mice were injected with tamoxifen (1 mg/mouse dissolved in corn oil) intraperitoneally once a day for ten consecutive days. Post tamoxifen injections, the genotypes of the mice were determined again to confirm the efficiency of inducible UBC-CreERT2. A designed primer pair 2 (5’ to 3’, forward: AGTTCCACTCGTCGTGGTGC; reverse: TGAATAGGGTCCAGGGGTGG) that can detect truncated *Qsox2* (QSOX2 KO) was utilized to identify the incised DNA fragment. RNA was extracted with the RNeasy Mini Kit (Qiagen, NL) (supplementary Fig. 1A). The quantity of RNA was assessed by Picodrop Spectrophotometer (Alpha Biotech, UK). Two μL of Random Hexamer Primer was added to each 11 μL RNA sample following the manufacturer’s instructions provided by Transcriptor First Strand cDNA Synthesis Kit (LifeScience, Germany). It is known that Tamoxifen is a selective estrogen receptor modulator and is widely applied to hormone-replacement therapy for breast cancer treatment. However, tamoxifen may abrogate estrogen-dependent hormonal regulation in normal male mice, and thereafter the disruption of reproductive endocrine leads to the reduction in fertility [32]. Therefore, caution is still needed on the effects of tamoxifen when interpreting our *QSOX2* knockout mouse model. Morphological changes of the reproductive organs and the diminished fertility in Ubc Cre-ERT; QSOX2^flox/△^ mice might be attributed to the combinational effects of tamoxifen and *Qsox2* deletion. Therefore, another mouse model without the potential interference of tamoxifen for validation is needed.

Generation of non-conditional *Qsox2* KO (no tamoxifen induction) was performed by pairing *Qsox2* ^flox/flox^ mice with SOX2-Cre mice. To enhance the efficiency of having *Qsox2* KO mice, *Qsox2* ^flox/−^ mice from the F2 were further paired. The PCR program (95ºC, 3 min., 95ºC, 30 s., 60ºC, 30 s., 72ºC, 1 min., 72ºC, 5 min, 35 cycles) was used for all of the samples to validate the genotype, and agarose gel electrophoresis was utilized for DNA separation and the later result visualization as described above. Primer pair 3 (5’ to 3’, forward: CAGCAGGGTCTCAGGTAGTTC; reverse: AGAACCAGGATGTGTGCCG) was used to validate the efficiency and specificity of the non-conditional *Qsox2* KO (supplementary Fig. 1B). To evaluate the effect of *Qsox2* KO on the reproductive capacity in mice, different genotypic combinations, i.e., *Qsox2* ^flox/−^ x *Qsox2* ^flox/−^, *Qsox2* ^flox/−^ x *Qsox2* ^−/−^, and *Qsox2* ^−/−^ x *Qsox2* ^−/−^ were also tested and mating outcomes were accessed. After determining the genotype of each mouse, the estrus cycle was monitored daily by vaginal smears as described above, and tissues, including ovaries, oviducts, and uterus, were collected for mRNA analyses and histologic examination.

### Histological examination and indirect immunofluorescence (IFA) staining

For histological examination, samples were fixed with 10% neutral buffered formalin overnight on a shaker and processed for paraffin embedding. Standard five-micron tissue sections were dewaxed and stained with hematoxylin–eosin (H&E). The slides were evaluated by the authors and a certified pathologist. To detect tissue and cellular localization of QSOX2 on the ovary, indirect immunofluorescent (IFA) staining was performed as described earlier [5, 6]. Five-micron paraffin-embedded tissue sections were deparaffinized and underwent antigen retrieval in 10 mM citrate buffer (pH 6.0) at 95ºC for 10 min (5 min/cycle). Tissue sections were permeabilized with 0.5% Triton-X 100 at room temperature (RT) for 10 min. The non-specific signal was minimized with 1% bovine serum albumin (BSA) for 60 min at RT. Anti-QSOX2 (1:200) was applied to the section overnight at 4ºC. After intense washing with filtered PBS, sections were subsequently incubated with goat anti-mouse or goat anti-rabbit Alexa-594 (1:150 diluted with 1% BSA) for 1.5 h at RT. Cell nuclei were counterstained with 4',6-diamidino-2-phenylindole (DAPI) (Vectashield H-1200, Vector Laboratories, Peterborough, UK), and slides were sealed with nail polish. Samples were examined with Olympus IX83 epifluorescent microscopy and analyzed with either ImageJ (NIH; http://rsb.info.nih.gov/ij/) or CellSens software (Tokyo, Japan). To clarify the potential contribution of the uterus to infertility, HE stain mosaic uterus images from wild-type control, heterozygous, and homozygous KO mice were shown in the supplementary Fig. 2.

### Next-generation sequencing (NGS) and bioinformatic analyses (RNA-seq)

The ovaries of three mice from wildtype *Qsox2* ^+/+^ and *Qsox2* ^−/−^ (*Qsox2* (QSOX2 KO) ovaries were collected for next-generation sequencing and bioinformatic analyses. Total RNA were extracted from tissues and subjected to cDNA synthesis and NGS library construction using the Universal Plus mRNA-Seq Library Preparation Kit with NuQuant (NuGEN Technologies, San Carlos, CA, USA). The quality and average length of the sequence library for each sample was assessed by the Bioanalyzer (Agilent Technologies, Santa Clara, CA, USA) and with the DNA 1000 kit, respectively. The indexed samples were pooled equimolarly and sequenced on Illumina NovaSeq 6000 (151 bases, paired-end reads). The quantification of raw reads was processed using CLC Genomics Workbench v.10 software. Adaptor sequences and bases with low quality or ambiguity were trimmed. The quality-screened reads were mapped to the Mus musculus genome GRCm38 (mm10) assembly from C57BL/6J strain using the CLC Genomics Workbench. The mapping parameters were as follows: mismatch cost 2, insertion cost 3, deletion cost 3, length fraction of 0.5, and similarity fraction of 0.8. The differential gene expression (DGE) between two or more conditions is based on the fold change of the FPKM value. The genes with twofold change were further analyzed. KEGG database [33, 34] was used in pathway enrichment analysis, and the pathway map was plotted by pathview [35] packages in R. Gene ontology (GO) enrichment was analyzed by GO-TermFinder [36].

### Statistical analysis

Data were presented as the mean ± standard deviation (SD). For statistical analysis, Fisher’s Exact test (for two groups comparisons) or one-way analysis of variance (ANOVA) followed by Tukey’s multiple comparisons test were carried out with GraphPad Prism (GraphPad Software, San Diego, CA, USA). Statistical differences were considered significant at *p*-value < 0.05.

### Supplementary Information

**Additional file 1: Supplementary Fig. 1.** PCR validation of mouse genotypes. (A) UBC-CreERT2 was used to generate single allele conditional knockout under 1 mg/mouse/day tamoxifen induction. Different primer sets were used to verify the presence of *Qsox2*^flox/+^ mice (primer pair#1) and the efficiency of Cre-recombinase induction of *Qsox2*^−/+^ mice (primer pair #2) (B) SOX2-Cre was used to generate systemic double allele *Qsox2* conditional knockout mice. PCR validation was conducted to identify wild-type *Qsox2*^+/+^, *Qsox2*^flox/flox^, and homozygous *Qsox2*^−/−^ (total knockout) mice. Red arrowheads indicated the corresponding base pair of the targeted genes.

**Additional file 2: Supplementary Fig. 2.** Histology of the uterus. The uterus of the wild-type control, heterozygous, and homozygous KO mice were examined for their histological abnormality to dissect the potential contribution of the uterus to the fertility outcomes of the mice.

## Data Availability

The datasets used and/or analyzed during the current study are available from the corresponding author upon reasonable request.

## Abbreviations

QSOX2: Quiescin sulfhydryl oxidase 2
QSOX: Quiescin Q6/FAD (flavin adenine dinucleotide)-dependent sulfhydryl oxidase
HPG: Hypothalamic-pituitary–gonadal
GnRH: Gonadotropin-releasing hormone
FSH: Pituitary follicle-stimulating hormone
LH: Luteinizing hormone
GCs: Granulosa cells
CL: Corpus luteum
ZP: Zona pellucida
IACUC: Institutional Animal Care and Use Committee
PBS: Phosphate-buffered saline
IP: Intraperitoneal
PCR: Poly chain reaction
KO: Knock out
IFA: Immunofluorescence
H&E: Hematoxylin-eosin
RT: Room temperature
BSA: Serum albumin
DAPI: 4',6-Diamidino-2-phenylindole
NGS: Next-generation sequencing
DGE: Differential gene expression
GO: Gene ontology
SD: Standard deviation
ANOVA: One-way analysis of variance
PCA: Principal component analysis
NMDS: Nonmetric multidimensional scaling
cAMP: Cyclic adenosine monophosphate
HIF-1: Hypoxia-inducible factor 1
MAPK: Mitogen‑activated protein kinase
PI3K: Phosphatidylinositol 3' -kinase
Mmp2: Matrix metallopeptidase 2
Mmp9: Matrix metallopeptidase 9
Pgr: Progesterone receptor
Ptgs2: Prostaglandin-endoperoxidase synthase 2
COCs: Cumulus-oocyte complexes
CREB: CAMP-response element-binding
MAPKAPK3: MAPK-activated protein kinase 3
FGF2: Fibroblast growth factor 2
PDGF: Platelet-derived growth factor
PDGF receptor A: PDGFRA
ANGPT1: Angiopoietin 1
TEK: Endothelial-specific tyrosine kinase
