# Supplementary figures and images for: Cre-LoxP and tamoxifen-induced deletion of ovarian quiescin sulfhydryl oxidase 2 showed disruption of ovulatory activity in mice
Source: J Ovarian Res. 2024 Mar 19;17:66. doi: 10.1186/s13048-024-01388-2 (PMC10949576; doi:10.1186/s13048-024-01388-2)

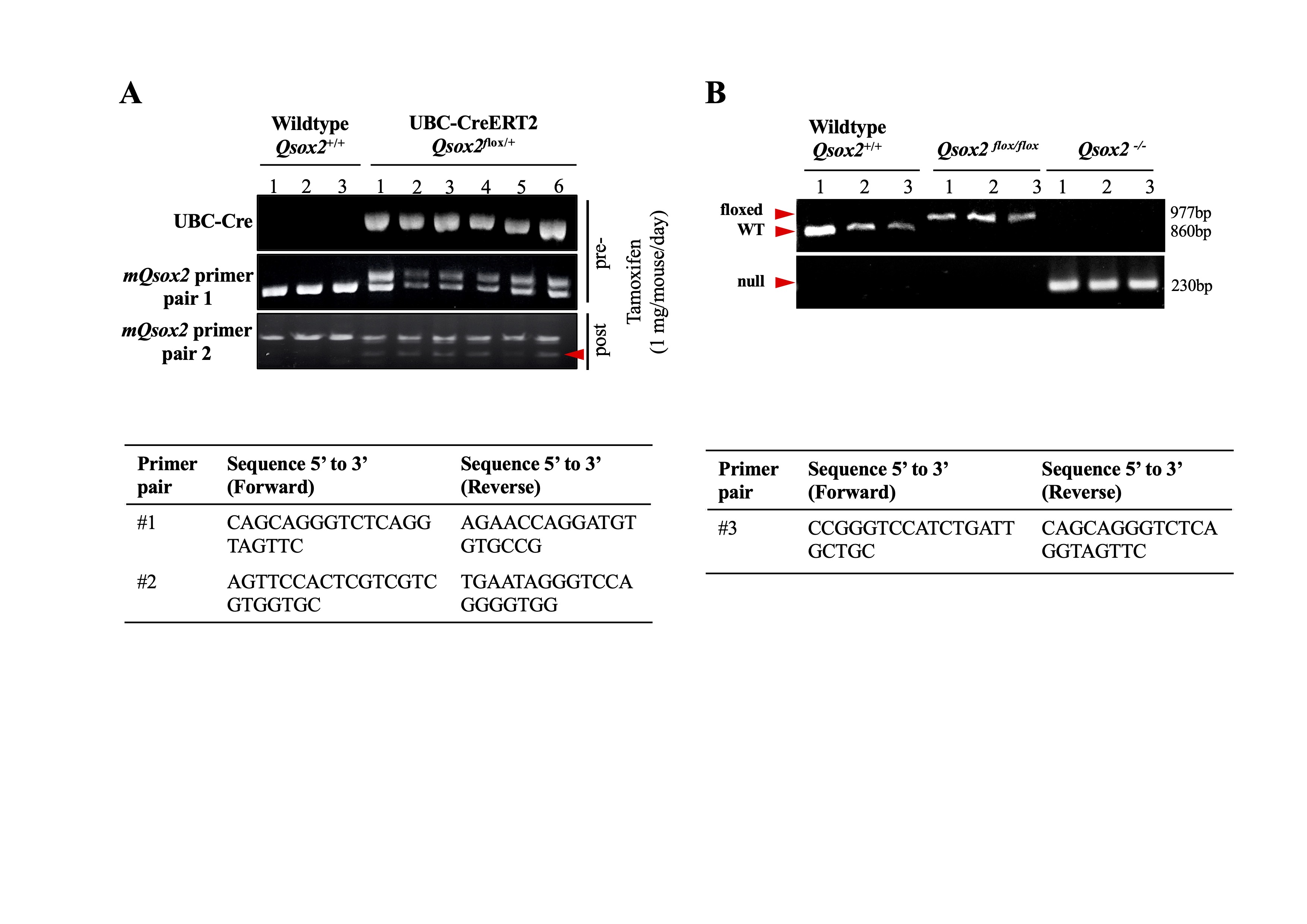

Supplement: Supplementary file 1 — Additional file 1: Supplementary Fig. 1. PCR validation of mouse genotypes. (A) UBC-CreERT2 was used to generate single allele conditional knockout under 1 mg/mouse/day tamoxifen induction. Different primer sets were used to verify the presence of Qsox2flox/+ mice (primer pair#1) and the efficiency of Cre-recombinase induction of Qsox2−/+ mice (primer pair #2) (B) SOX2-Cre was used to generate systemic double allele Qsox2 conditional knockout mice. PCR validation was conducted to identify wild-type Qsox2+/+, Qsox2flox/flox, and homozygous Qsox2−/− (total knockout) mice. Red arrowheads indicated the corresponding base pair of the targeted genes. [file 13048_2024_1388_MOESM1_ESM.jpg]

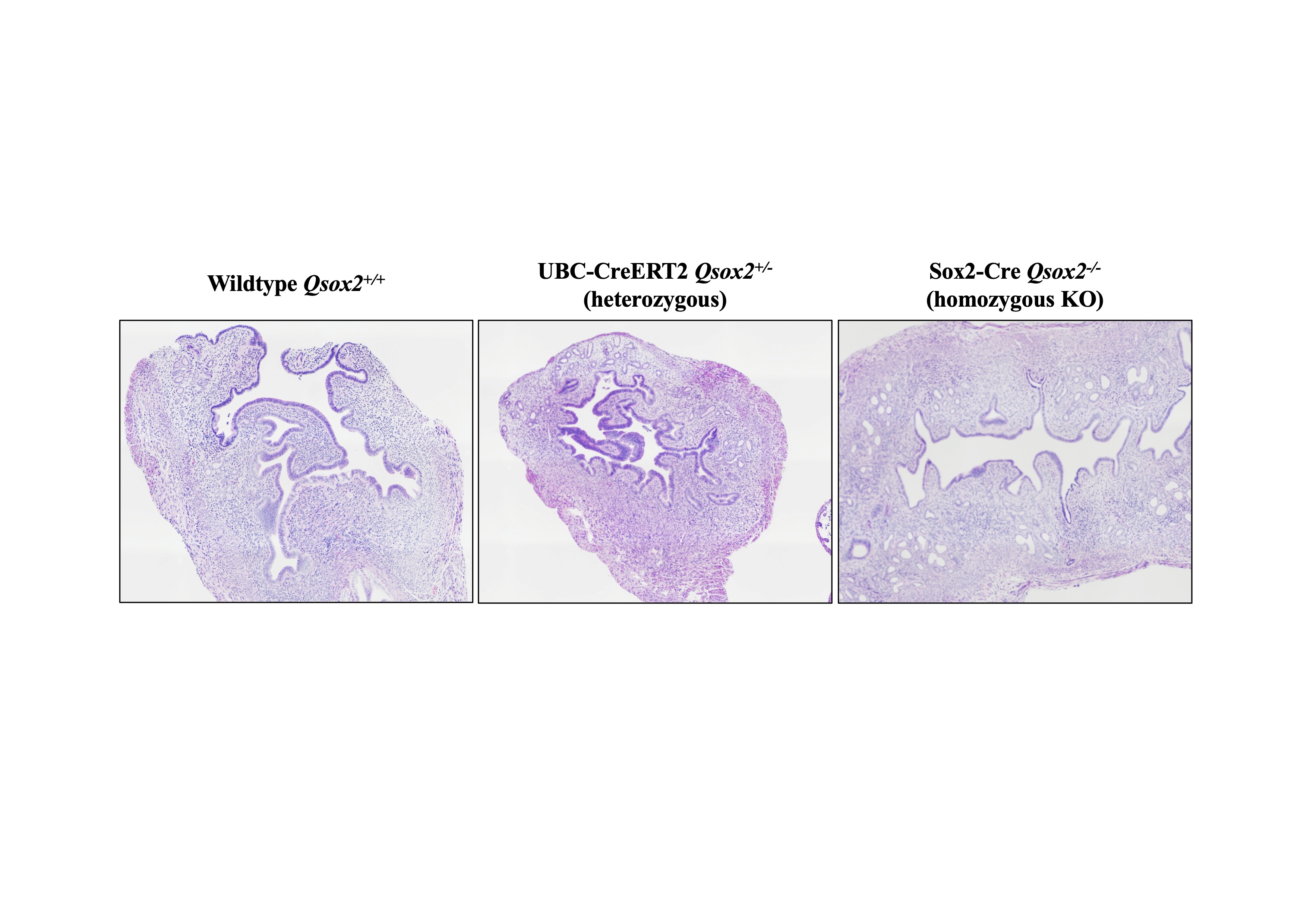

Supplement: Supplementary file 2 — Additional file 2: Supplementary Fig. 2. Histology of the uterus. The uterus of the wild-type control, heterozygous, and homozygous KO mice were examined for their histological abnormality to dissect the potential contribution of the uterus to the fertility outcomes of the mice. [file 13048_2024_1388_MOESM2_ESM.jpg]
